# The Controversial Role of TGF-β in Neovascular Age-Related Macular Degeneration Pathogenesis

**DOI:** 10.3390/ijms19113363

**Published:** 2018-10-27

**Authors:** Gian Marco Tosi, Maurizio Orlandini, Federico Galvagni

**Affiliations:** 1 Ophthalmology Unit of the Department of Medicine, Surgery and Neuroscience, University of Siena, Siena 53100, Italy; gmtosi18@gmail.com; 2 Department of Biotechnology, Chemistry and Pharmacy, University of Siena, Siena 53100, Italy

**Keywords:** neovascular age-related macular degeneration (nAMD), TGF-β, angiogenesis, choroidal neovascularization (CNV)

## Abstract

The multifunctional transforming growth factors-beta (TGF-βs) have been extensively studied regarding their role in the pathogenesis of neovascular age-related macular degeneration (nAMD), a major cause of severe visual loss in the elderly in developed countries. Despite this, their effect remains somewhat controversial. Indeed, both pro- and antiangiogenic activities have been suggested for TGF-β signaling in the development and progression of nAMD, and opposite therapies have been proposed targeting the inhibition or activation of the TGF-β pathway. The present article summarizes the current literature linking TGF-β and nAMD, and reviews experimental data supporting both pro- and antiangiogenic hypotheses, taking into account the limitations of the experimental approaches.

## 1. Introduction

Age-related macular degeneration (AMD) is considered one of the main causes of severe vision loss in elderly people. AMD has two stages: early and late AMD. Early AMD is characterized by changes in the retinal pigment epithelium (RPE) and the appearance of drusen, hyaline and fat pathological deposits that form between the RPE and Bruch’s membrane. Most people do not experience vision loss at this stage, but a minority of patients can progress to late AMD. Late AMD can be divided into two subtypes: geographic atrophy, or dry AMD, and the neovascular form (nAMD). The latter is the least frequent, but is responsible for the most severe vision loss. nAMD is characterized by choroidal neovascularization (CNV) under the macula that is driven by a cascade of proinflammatory and proangiogenic responses originating from damage of the outer retinal cells and RPE. New abnormal blood vessels first proliferate under the RPE and Bruch’s membrane and then invade the subretinal space, leading to subretinal hemorrhages, exudative lesions, retinal detachment, and the formation of fibrous scarring in the late stage of nAMD, with consequent permanent reduction in macular function and vision. Among proangiogenic agents, vascular endothelial growth factor-A (VEGF-A) plays a central role in promoting CNV, and intravitreal injection of anti-VEGF-A agents is the current treatment of choice for nAMD. Unfortunately, most patients require frequently repeated injections and regular long-term follow-up, with a significant percentage of them showing resistance to anti-VEGF-A therapies [1]. Thus, there is a need for investigating of alternative targets and intervention strategies, which could be associated with anti-VEGF treatment. The pleiotropic transforming growth factor-beta (TGF-β) cytokines have been extensively studied and have emerged as important players in angiogenesis. In the context of AMD treatment, both inhibition and induction of the TGF-β signaling pathway has been recently advocated as an additional tool. This paper reviews the literature concerning the role of TGF-β in the pathogenesis of nAMD, with emphasis on controversial and unresolved aspects.

## 2. TGF-β Synthesis and Signaling Pathway

In mammals, the TGF-β family consists of three members, TGF-β1, TGF-β2 and TGF-β3, which are encoded by three independent genes. The three different mature isoforms are characterized by a high degree of amino acid sequence identity (71.4–79.5%) and similarity (85.7–92%), with the highest levels shown by TGF-β2 and TGF-β3 [2].

**Figure 1:**
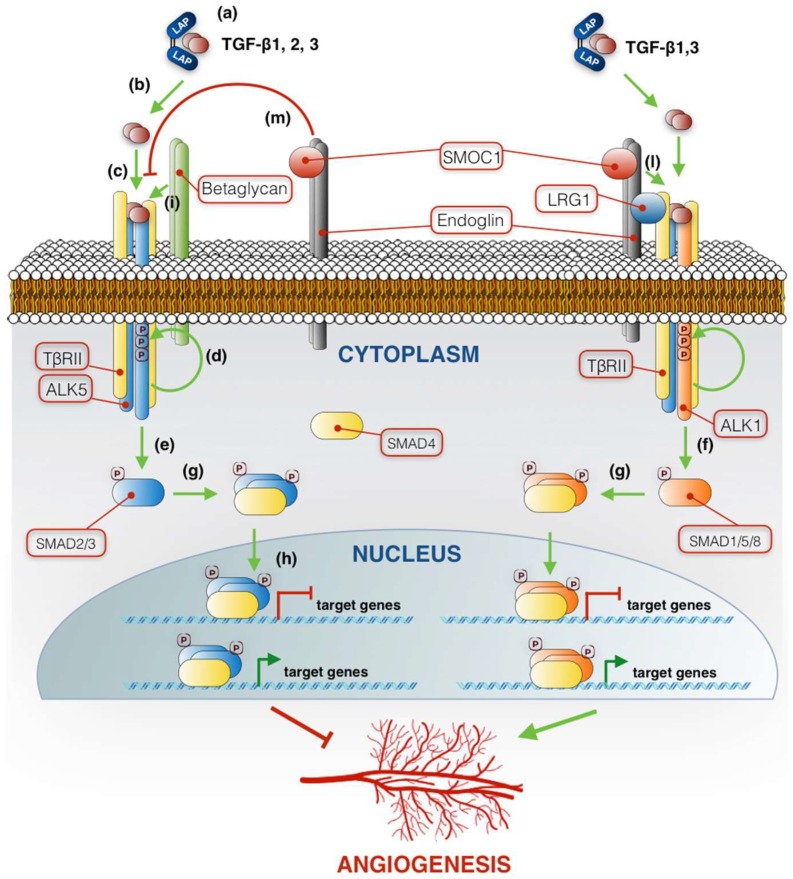
Schematic representation summarizing the transforming growth factors-beta (TGF-β) receptor complexes and signaling pathway in endothelial cells. The green arrowhead lines represent positive crosstalk interactions and steps. Red flat-ended lines indicate inhibition. In the nucleus, the green arrowhead and red flat-ended lines on the DNA represent activation and inhibition of gene expression, respectively. (**a**) LAP-TGF-β latent complex. (**b**) Release of TGF-β from the complex with LAP. (**c**) TGF-β binding to the heterotetrameric receptor complex. (**d**) TβRII phosphorylation of type I receptor ALK5. (**e**) SMAD2/3 phosphorylation by ALK5. (**f**) SMAD1/5/8 phosphorylation by ALK1. (**g**) Dimerization of R-SMADs with SMAD4. (**h**) Translocation of the R-SMAD/SMAD4 complex into the nucleus and binding to regulatory sequences. See text for details.

TGF-βs are synthesized as propeptides of 390 (TGF-β1) or 412 (TGF-β2 and TGF-β3) amino acids, composed of two regions: latency-associated peptide (LAP) and TGF-β. The propeptide is glycosylated on LAP, cleaved in the Golgi apparatus by the convertase FURIN, separating LAP from TGF-β, and secreted as heterotetrameric LAP-TGF-β latent complex, which is not biologically active because it is unable to bind to receptors (Figure 1a) [3,4]. In order to exert its biological effects, TGF-β needs to be released from the complex with LAP as a disulfide-linked 25 kDa dimer by several possible factors, such as pH, proteases or reactive oxygen species (Figure 1b) [5]. Active TGF-β binds to specific heterotetrameric transmembrane Ser/Thr kinase receptor complexes containing two of each of type I and type II subunits (Figure 1c). Among the five existing type II receptors (ActR-II, ActR-IIB, TβRII, BMPR-II and AMHR-II), TGF-βs bind specifically TβRII, which promotes recruitment of either TβRI (also named activin receptor-like kinase 5, ALK5) or ALK1, two of seven structurally related type I receptors (ActR-I, ActR-IB, TβRI, ALK1, ALK7, BMPR-IA and BMPR-IB) [6]. Having formed the tetrameric receptor complex, the constitutively active cytoplasmic kinase domain of TβRII phosphorylates the glycine/serine-rich domain of type I receptors at multiple serine and threonine sites, thus resulting in activation of the type I receptor kinase (Figure 1d). In the canonical signaling pathway, activated type I receptors recruit and serine-phosphorylate the transcription factors R-SMADs (receptor-regulated SMADs). More specifically, ALK5 induces SMAD2 and SMAD3 phosphorylation (Figure 1e), while ALK1 transduces the signal through SMAD1, SMAD5 and SMAD8 phosphorylation (Figure 1f) [7,8]. Phosphorylation of R-SMADs results in their dimerization and association with a common SMAD (co-SMAD or SMAD4) (Figure 1g). The R-SMAD/co-SMAD complex translocates into the nucleus, where it binds to regulatory sequences, the so-called SMAD binding elements, within promoters and enhancers of target genes to activate or repress their transcription by recruiting a variety of coactivators or corepressors to the chromatin (Figure 1h) [9].

### 2.1. TGF-β Family Signaling in Angiogenesis

In endothelial cells (ECs), both type I receptors ALK1 and ALK5 are expressed, and the TGF-β signaling pathway results in activation of both classes of SMAD proteins [8]. Furthermore, ALK5 is necessary for efficient transduction of ALK1 [10]. The TGF-β/ALK5/SMAD2/3 and TGF-β/ALK1/SMAD1/5/8 axes exert opposite effects on angiogenesis. In fact, it has been described that ALK1 promotes EC migration and proliferation, while ALK5 inhibits these processes and is essential for maintaining the integrity of mature vessels (Figure 1) [8,10,11,12]. These conflicting effects of TGF-β on angiogenesis are due to different contingent factors, such as: ALK1 expression level, regulation of type I and type II receptor signaling by soluble ligand-binding proteins and by accessory type III receptors (TβR III), and different affinity of the three TGF-β family members to receptors and co-receptors. ALK1 is highly expressed in the embryo vasculature, becoming less detectable in the quiescent endothelium during adult life. However, ALK1 expression rapidly increases in response to angiogenic stimulation [13,14,15], and the consequent altered balance between ALK1 and ALK5 pathways promotes the proangiogenic effect of TGF-β [10]. 

TGF-β1 and TGF-β3 bind receptors and signal in a similar manner, interacting with TβRII independently of TβRI, which is subsequently recruited to the receptor complex [16,17]. On the contrary, TGF-β2 binds very weakly to TβRII alone and requires betaglycan, a type III TGF-β co-receptor (TβRIII), to activate the complex (Figure 1i) [18,19]. Betaglycan is a membrane-anchored proteoglycan that binds and presents all three TGF-β isoforms to TβRII, potentiating their effects, although it is essential only for TGF-β2. Endoglin, also named CD105, is the other known TβRIII and is predominantly expressed in ECs, where its expression level increases during angiogenesis [20,21]. Endoglin shows 63% homology to betaglycan [22], interacts as a homodimer with TβRII, ALK1 and ALK5 [23,24], and binds to TGF-β1 and TGF-β3, but only when in association with TβRII [25,26]. Interestingly, similarly to what was observed for TβRII, endoglin does not interact with TGF-β2 [25]. In complex with ALK1, ALK5 and TβRII, endoglin is necessary for the activation of TGF-β-dependent ALK1 signaling (Figure 1l) [27] and indirectly reduces the TGF-β/ALK5/SMAD2/3 pathway (Figure 1m), thereby promoting proliferation of ECs and blocking TGF-β-induced growth arrest [28,29]. 

Recently, two soluble regulators of TGF-β signaling have been identified: secreted modular calcium-binding protein 1 (SMOC) and leucine-rich alpha-2-glycoprotein 1 (LRG1) [30,31]. SMOC1 is a matricellular protein [32] and, as such, it is secreted into the extracellular environment, but it does not play a structural role in the extracellular matrix (ECM). SMOC1 is highly expressed in proliferating ECs and its expression is increased in hypoxia [30]. The study by Awwad and colleagues suggests that SMOC1 promotes EC proliferation and angiogenesis by binding to endoglin and acting as a negative regulator of ALK5 signaling (Figure 1m) [30]. LRG1 is a secreted glycoprotein and, in the presence of TGF-β1, is mitogenic to ECs and promotes angiogenesis. It has been demonstrated that LRG1 binds ALK5, TβRII and endoglin and, similarly to SMOC1, elicits its proangiogenic effects by switching TGF-β signaling towards the ALK1 axis [31].

### 2.2. TGF-β Expression in the Human Eye

**Figure 2:**
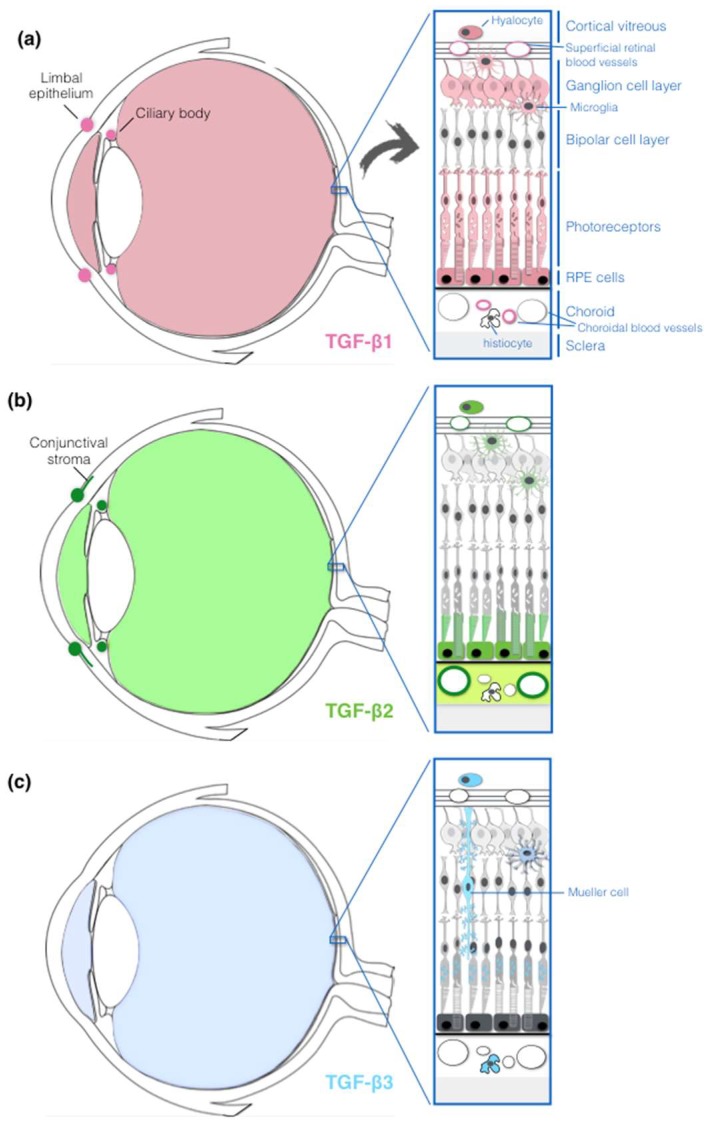
Drawing of a section through the human eye with a schematic enlargement of the retina layers. The TGF-β1 (**a**), TGF-β2 (**b**) and TGF-β3 (**c**) expression in human eye structures and cells are indicated by different colors. See text for details.

All three TGF-β isoforms were detected in aqueous and vitreous humor of the human eye (Figure 2), and it was determined that approximately 87% of the vitreal TGF-β is in the latent form [33,34,35,36,37,38,39]. Although different detection methods were used to measure TGF-β concentrations, all studies agree that TGF-β2, both active and latent, is the predominant isoform in normal aqueous and vitreous humor, and that active TGF-β1 is not detectable [33,37,39,40]. In human aqueous humor, the ratio among the three active isoforms was estimated to be 1:0.4:0 for β2:β3:β1, respectively [39]. Immunolocalization of TGF-β isoforms in the anterior segment of the eye has evidenced that TGF-β1 is found in the superficial limbal epithelium, in the proximal portion of ciliary processes and in the wall of blood vessels within the ciliary body (Figure 2a) [41]. TGF-β2 is found in the limbal epithelium, in the connective tissue of conjuntival stroma, in the ciliary body muscle and stroma, in the wall of blood vessels within the ciliary body, and in fibroblast-like cells in the ciliary processes (Figure 2b). On the contrary, no immunolabeling for TGF-β3 was detected in any structure of the anterior eye [41]. Even in the posterior segment of the human eye, TGF-β isoforms are distributed heterogeneously. TGF-β1 can be detected in the endothelium of choriocapillaries, microglia, photoreceptors, ganglion cells, smooth muscle cells (SMCs) and pericytes of superficial retinal blood vessels, and vitreous hyalocytes (Figure 2a). TGF-β2 is localized in the connective tissue of large choroidal vessels, choroidal stroma, outer segment of photoreceptors, microglia, SMCs and pericytes of superficial retinal blood vessels, and vitreous hyalocytes (Figure 2b). TGF-β3 is found in choroidal histiocytes, microglia, Müller glia cells, vitreous hyalocytes and within the mitochondria of photoreceptors (Figure 2c) [42,43]. Moreover, TGF-β1 and β2, but not β3, are expressed by human RPE cells [44,45,46,47,48]. The predominant expression of TGF-β2 over TGF-β1 and TGF-β3 observed in vitreous and aqueous humor was confirmed also in human RPE cells [44,45,47,48] and in monkey ocular tissues, such as the retinal photoreceptor outer segment layer, the RPE-Bruch’s membrane-choroid (RPEBC) complex, RPE cells in situ, and cultured RPE cells [34]. Moreover, the same study demonstrated that TGF-β2 tissue concentration is 10-fold higher in the RPEBC complex than in neural retina, and that cultured RPE cells release a high amount of TGF-β2, versus undetectable levels of β1, suggesting that this cell type is a source of the TGF-β2 detected in the RPEBC complex. The prevalent role of TGF-β2 in the eye is also confirmed by the observation that lack of TGF-β2, but not TGF-β1 or TGF-β3, perturbs mouse embryonic morphogenesis of the eyes [49,50].

## 3. Evidence for Proangiogenic Function of TGF-β in nAMD

**Figure 3:**
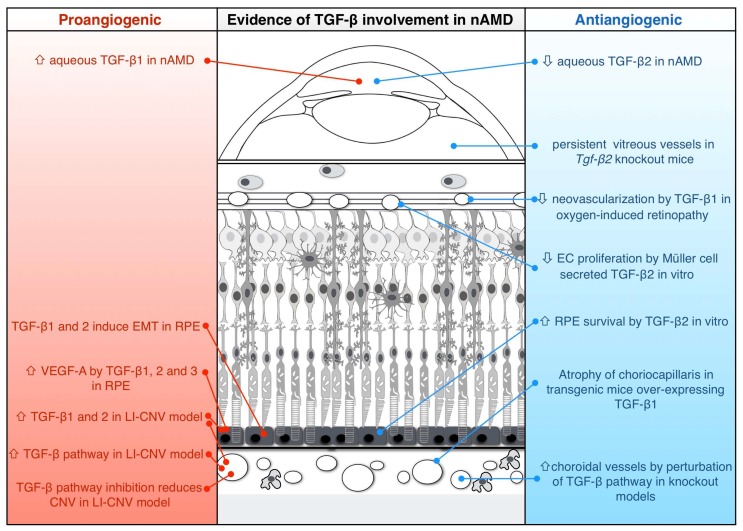
Schematic representation summarizing the evidence of a proangiogenic (left column) and antiangiogenic (right column) role played by TGF-β in nAMD. Arrows pointing up indicate upregulation, and arrows pointing down indicate downregulation. Red and blue lines connect each experimental evidence with the related site in the eye. Where not specified, the experimental procedures adopted do not permit one to unequivocally identify the TGF-β type involved. EC: endothelial cells; EMT: epithelial-to-mesenchymal transition; CNV: choroidal neovascularization; LI-CNV: laser-induced CNV; RPE: retinal pigment epithelium. See text for details.

Several studies support a promoting role of TGF-β in nAMD (Figure 3). The proangiogenic function of TGF-β in nAMD could be direct, through the stimulation of choroidal EC proliferation [31], or indirect, through the induction of VEGF-A secretion by RPE cells or of the macrophage-mediated inflammation [51]. VEGF-A secretion by RPE cells plays an important role in retinal and choroidal neovascularization and it is promoted in vitro by various stimuli, including hypoxia [52], physical disruption of cell–cell contact [53], complement components C3a and C5a [54] and inflammatory cytokines such as interleukine-1, interferon-γ, TNF-α [55], and TGF-β1, TGF-β2 and TGF-β3 [56,57].

The strongest evidence for a proangiogenic function of TGF-β in nAMD is provided by in-vivo experiments of laser-induced CNV (LI-CNV) in rodents [58]. The LI-CNV is a gold-standard animal model for nAMD research. By targeted laser injury of the RPE and Bruch’s membrane, the procedure induces choroidal angiogenesis, similarly to what is observed in nAMD. Immunofluorescent staining and Western blot analysis demonstrated that TGF-β1 protein expression is upregulated during LI-CNV development in mice [35,59], and both TGF-β1 and TGF-β2 mRNAs (the latter being expressed more prominently) were upregulated after laser treatment, especially in the endothelium of neovascular regions [60]. Moreover, during LI-CNV, inhibition of the TGF-β pathway, by intraperitoneal injection of LY2157299 (an ALK5 and TβRII inhibitory compound) or intravitreal injection of decorin (a TGF-β binding and inhibitory protein), reduced the levels of VEGF-A expression and CNV formation in the RPEBC complex [59]. Similarly, Recalde and colleagues showed a reduction of LI-CNV lesions in rats after administration of peptides against TGF-β, including TGF-β2, either before or during early stage of CNV development [61,62]. However, the laser-induced animal model represents an acute injury and inflammation, mimicking only the neovascular component of disease, and consequently is unable to recapitulate the complex sequence of events leading to the development of CNV in patients with nAMD, such as long-standing senescent degeneration and chronic inflammation. Anatomic discrepancies exist as well, since mice and rats lack a macula, and the laser coagulation causes significant damage to the overlying neural retina to a greater extent than in human nAMD [63].

The proangiogenic function of TGF-β in nAMD is also supported by the circumstantial evidence that both vitreous and aqueous TGF-β1 concentrations are increased in patients as compared to controls, even though it must be remembered that TGF-β1 is the least abundant isoform in the human eye [35,38]. These studies are in agreement with the observations that TGF-β expression is significantly increased in the RPE of human maculae with nAMD [46,64]. However, in the latter studies it is not clear which TGF-β isoform was specifically detected, because, in the immunolocalization experiments, the authors used a “panspecific” polyclonal antibody (AB-100-NA, from R&D Systems) raised in rabbit against a mixture containing recombinant human TGF-β1 and porcine TGF-β2, without any specification of isoform affinity or evidence for human TGF-β2 recognition. Moreover, it has been observed that TGF-β1, TGF-β2 and TGF-β3 are potent inducers of VEGF-A mRNA level and protein secretion in human primary RPE (hpRPE) cells cultured in vitro [56,57].

Finally, the LRG1- or SMOC1-mediated switch of TGF-β signaling towards the ALK1 axis could be an important actor in promoting pathological angiogenesis in the eye. The SMOC1 role in angiogenesis of the eye was investigated by the model of postnatal development of intraretinal vasculature. In contrast to humans, mouse pups have an immature intraretinal vasculature, whose development goes on postnatally. In SMOC1^+/−^ mice, this process was significantly delayed in comparison to SMOC^+/+^ mice, suggesting a positive involvement of the TGF-β/ALK1/SMAD1/5/8 axis [30]. It must be underlined, however, that this model is quite far from what really happens in the development of AMD-related CNV, and further, more targeted studies are needed to clarify the role of SMOC1 in this context. Conversely, the data regarding LRG1 are more complete. *Lrg1* transcript was upregulated in vessels of both the retina and RPE/choroid following LI-CNV. Moreover, laser-induced neovascular response was reduced in *Lrg1*^−/−^ mice or after intravitreal injection of LRG1 neutralizing antibody [31]. However, even in this case, the confirmation of LRG1 involvement in nAMD development in humans is missing.

## 4. Evidence for Antiangiogenic Function of TGF-β in nAMD

As described before, TGF-β is normally expressed in several eye structures and is involved in important physiological processes. In vitro, it has been demonstrated that Müller glial cells inhibit proliferation of retinal ECs by TGF-β2 secretion [65], RPE-derived TGF-β leads microglia to an anti-inflammatory phenotype [66,67], and TGF-β2 supports RPE cell survival on aged and AMD Bruch’s membrane [68]. 

Strong evidence for antiangiogenic function of TGF-β in nAMD derives from in-vivo studies that are not based on LI-CNV (Figure 3). In a transgenic mouse model, ocular overexpression of active TGF-β1 induced the atrophy of choriocapillaris without any sign of CNV [69,70]. Specularly, in the developing eyes of mice lacking *Tgf-β2* (Tgfb2^−/−^), persistent vitreous vessels could be detected [50]. Moreover, the induced conditional deletion of TβRII in the entire eye or in the vascular endothelium of the eye, but not in RPE, caused an increased retinal expression of VEGF-A, the development of CNV, and the induction of other phenotypic characteristics of nAMD [71]. In a mouse model of oxygen-induced retinopathy, intraperitoneally injected human placental amniotic membrane-derived mesenchymal stem cells migrated into the retina and suppressed excessive neovascularization by TGF-β1 expression [72]. In a rat model mimicking early AMD stages, intravitreal injection of human recombinant TGF-β1 prevented retinal insult induced by intravitreal injection of amyloid-beta 1–40 fragments, a constituent of drusen [73,74,75].

In humans, contrary to what is observed for TGF-β1, aqueous levels of active TGF-β2 are lower in nAMD patients as compared to controls, even after anti-VEGF-A treatment, while TGF-β3 expression remains unchanged [39]. This observation is made more significant by the fact that TGF-β2 is the predominant isoform in the human eye and seems to be more specific for the activation of SMAD2/3 (antiangiogenic) transcriptional response, because of its dependency on betaglycan for receptor binding and its inability to bind endoglin. However, even though it is generally accepted that cytokine levels in the aqueous samples reflect the intraocular concentrations [76], the measure of TGF-β2 and TGF-β3 vitreous concentrations and their immunolocalization analysis within human CNV membrane are missing to clarify their role in nAMD, also considering that vitreous TGF-β2 and TGF-β3 concentrations are augmented in other ocular diseases [77,78,79,80]. The antiangiogenic function of TGF-β is also supported by the observation that SMAD2 is phosphorylated in the EC nuclei of normal choroidal vessels but not of CNV membranes from naïve nAMD patients, and that the TGF-β activity is reduced in nAMD aqueous humor samples as compared to controls [39].

## 5. TGF-β Signaling in RPE

ECs are not the only TGF-β targets called into question for AMD. Human RPE cells express ALK5 and TβRII, and respond to TGF-β stimulation [81]. Moreover, RPE cells secrete TGF-β2 and this secretion is increased when RPE cells lose polarity in both confluent and subconfluent culture conditions in vitro [48]. TGF-β2 enhances survival of hpRPE cells on submacular Bruch’s membrane of aged and AMD donor eyes [68], and reduces the proliferation rate of hpRPE cells [82]. In AMD patients, it is commonly observed that at sites of CNV, the RPE loses its barrier function and transdifferentiates from its epithelial structure to a mesenchymal phenotype in a process called epithelial-to-mesenchymal transition (EMT) [83,84]. TGF-β signaling has been reported to be a potent mediator of RPE EMT both in vitro and in a transgenic mouse model carrying ocular overexpression of active TGF-β1 [69,85,86,87]. It has been demonstrated that in an RPE cell line (ARPE-19), TGF-β induced the expression of a classical mediator of EMT, the transcription factor SNAI1. SNAI1 promoted the decrease of E-cadherin and zona occludens-1 expression, two cell–cell junction proteins playing a crucial role in the formation and maintenance of epithelial barrier. SNAI1 also mediated the increase of fibronectin and α-smooth muscle actin expression, and, consequently, the migratory activity of RPE cells [88]. As further confirmation of this, it was reported that TGF-β1 led to an increase in expression of mesenchymal markers in stem cell-derived RPE cells, along with a decrease in expression of epithelial markers [89], and TGF-β2 promoted ARPE-19 cell invasion into collagen by mediating the expression urokinase-type plasminogen activator, a serine protease involved in tissue remodeling and cell migration [90]. Nevertheless, TGF-β2 was unable to initiate EMT in primary porcine RPE isolated as sheets, cultured in vitro on lens capsules, and as such, maintaining cell–cell contact [91]. Taken together, these observations on TGF-β signaling in RPE suggest a scenario in which TGF-β could play opposite effects on RPE in the development or progression of nAMD. Physiological low levels of autocrine TGF-β2 could be necessary for RPE homeostasis. Loss of epithelial integrity and RPE polarity seem to induce an increased secretion of TGF-β2, which in turn promotes EMT, with RPE cells losing normal cell shape and their epithelial function, and exhibiting migratory behavior.

## 6. TGF-β and Subretinal Fibrosis

By histological analysis of CNV membranes obtained performing submacular surgery, it has been demonstrated that CNV can often culminate in subretinal scarring (fibrosis) in approximately half of all treated eyes within two years of anti-VEGF treatment [92,93]. Importantly, it is the scarring response that irreversibly damages photoreceptors. This process is a consequence of excessive wound healing response to tissue damage and is characterized by proliferation and/or infiltration of various types of cells, such as RPE cells, glial cells, fibroblasts, myofibroblast-like cells and macrophages, and substantial remodeling of the ECM [93,94].

TGF-β has often been described to be involved in several aspects of subretinal fibrosis. In ARPE-19 cells, TGF-β2 promoted cell invasion into a collagen gel and induced the expression of collagen type I and fibronectin [90,95,96,97], two of the most prominent ECM components in subretinal fibrosis that play important roles in cell migration [94,98]. Moreover, in hpRPE cells, TGF-β induced secretion of PDGF that can directly induce their proliferation and activate fibroblast cells [82,99].

The involvement of TGF-β in this process has been demonstrated in animal models obtained by laser photocoagulation and subsequent subretinal injection of macrophage-rich peritoneal exudate cells. Levels of active TGF-β1 and TGF-β2, but not TGF-β3, were strongly upregulated in mice with subretinal fibrosis in comparison to control mice, and intraperitoneal injection of TGF-β-neutralizing antibodies resulted in reduced subretinal fibrosis areas [100]. Finally, in the same in-vivo model, similar results have been obtained by inhibition of cyclooxygenase-2 with the consequent downregulation of VEGF-A and TGF-β2 [101].

## 7. TGF-β Signaling in Retinal Neuronal Cells

As described for RPE cells, the TGF-β pathway has an ambivalent effect on retina neuronal cells. In TβRII-deficient mouse, a significant increase of apoptosis in retinal neurons was described during embryonic and postnatal development without affecting their proliferation. Moreover, treatment with TGF-β2 reduced death of retinal ganglion cells in dissociated retinal cell cultures, and this effect was blocked by inhibiting the phosphorylation of SMAD3 [102]. In a mouse model of conditional deletion of TβRI in retinal neurons, TGF-β signaling seemed to be required for the synthesis of chondroitin sulfate proteoglycans and the consequent retinal attachment to RPE [103]. On the other hand, retinal hypercellularity was reported in TGF-β2 knockout mice, suggesting the positive role of TGF-β2 in programmed cell death during retinal development [49]. This observation was confirmed by application of a neutralizing anti-TGFβ1/2/3 antibody or exogenous recombinant TGF-β1 to retinal cultures, which resulted in a significant decrease or increase, respectively, in the number of apoptotic ganglion cells [104]. However, all these studies focus on developing retina, and experimental data addressing the role of the TGF-β pathway in retina neuronal cells in the context of early or late AMD are missing. 

Several studies suggest that TGF-β1 has a neuroprotective role in the brain against a wide variety of death-inducing insults, including hypoxia/ischemia, amyloid-beta, and oxidative damage. This effect can be hypothesized also for retinal neuronal cells, considering that many protein and lipid constituents of drusen are similar to those found in deposits characteristic of other age-related degenerative disorders such as Alzheimer’s disease and other amyloid diseases. This link between AMD and Alzheimer’s disease, and the neuroprotective role of TGF-β, are also supported by the observation that retinal and circulating miRNAs miR-27a and miR-146a are upregulated in plasma of AMD patients and in the retina of amyloid-beta-injected rats, as well as in Alzheimer’s disease patients [105]. Interestingly, both these miRNAs are known to downregulate the TGF-β signaling pathway by targeting SMAD factors [106,107].

## 8. Conclusions

The current vascular-directed treatments for nAMD target the VEGF-A pathway to induce the quiescence of the vasculature network. However, they are only partially effective. Therefore, inhibition of the TGF-β pathway has been advocated as an additional treatment for nAMD, but further efforts are necessary to clarify its controversial involvement in nAMD pathogenesis, and several aspects have to be taken into account before proceeding to TGF-β pathway-targeted therapy:

1. In animal models resembling early stages of nAMD, TGF-β was shown to be antiangiogenic, while proangiogenic in those resembling acute or late disease stages. Similarly, TGF-β could play opposite roles at early, intermediate or late stages in humans. 

2. The TGF-β superfamily comprises over 30 members including activins, nodals, bone morphogenetic proteins (BMPs), and growth and differentiation factors. Their signaling converges at the level of receptors and/or of SMAD factors, and induces differential effects on the angiogenic process [108]. The involvement of some of these factors, such as BMP9, BMP10 or BMP4, in nAMD pathogenesis, could complicate the scenario [109,110,111,112]. 

3. The role of co-receptor molecules, such as LRG1 and SMOC1, that switch the TGF-β response from anti- to proangiogenic, has to be deepened in the context of nAMD, and could represent more attractive and specific targets (even considering that basal levels of TGF-β are necessary for the homeostasis of some eye tissues) [103,113,114].

Finally, the recent results from a collaborative genome-wide association study, examining more than 17,100 advanced AMD cases and 60,000 controls of European and Asian ancestry, have implicated *TGFBR1*, the gene coding for ALK5, in AMD pathogenesis due to an informative SNP within the sixth intron of the gene (*p* < 5 × 10^−8^) [115]. However, this observation is not actually informative about the TGF-β pathway’s role in AMD. This is because the SNP effect on *TGFBR1* transcription and/or splicing is unknown. Future studies taking into account this aspect will be of great interest. 

## Abbreviations

TGF-β	transforming growth factor-beta
nAMD	neovascular age-related macular degeneration
CNV	choroidal neovascularization
LI-CNV	laser-induced CNV
TβRII	TGF-β type II receptor
ALK	activin receptor-like kinase
SMAD	small mother against decapentaplegic
VEGF-A	vascular endothelial growth factor-A
RPE	retinal pigment epithelium
EC	endothelial cell
hpRPE	human primary RPE
